# What is the attitude towards and the current practice of information exchange during self-medication counselling in German community pharmacies? An assessment through self-report and non-participant observation

**DOI:** 10.1371/journal.pone.0240672

**Published:** 2020-10-14

**Authors:** Jasmin Mina Seiberth, Katharina Moritz, Nagihan Kücükay, Susanne Schiek, Thilo Bertsche

**Affiliations:** 1 Department of Clinical Pharmacy, Institute of Pharmacy, Faculty of Medicine, Leipzig University, Leipzig, Germany; 2 Drug Safety Center, University Hospital Leipzig and Leipzig University, Leipzig, Germany; The University of Sydney School of Pharmacy, AUSTRALIA

## Abstract

**Background:**

Guidelines encourage relevant information exchange between pharmaceutical staff and patients during self-medication consultation. Thereby, assessing the patient’s situation and providing information is crucial for patient safety. So far, limited studies have investigated this information exchange, particularly in Germany. We aimed to assess the attitude towards and the current practice of guideline-recommended information exchange in German community pharmacies.

**Methods:**

In total, twelve guideline-recommended parameters were predefined for gathering patient-related information and for the provision of information. These information exchange parameters were evaluated in two parts: Firstly, in a self-report of pharmaceutical staff via an online questionnaire to assess the reported importance, difficulty and frequency of the parameters as well as barriers to their implementation; secondly, in a non-participant observation in five pharmacies to evaluate the actual consultation practice.

**Results:**

In the self-report, all parameters were rated by more than 76% of 1068 participants as important. ‘Concurrent medication’ was determined to be the most difficult parameter to address (54%). All parameters of information gathering were rated to be addressed during routine counselling by at least 70% of the respondents. Parameters of information provision were all rated to be addressed by at least 45%. ‘Lack of patient’s interest’ was identified as the most frequent barrier to appropriate counselling (84%). During the observation, the information gathering parameters were each addressed between 8 to 63% in the consultations, parameters of information provision between 3 to 34%.

**Conclusion:**

Despite broad acceptance, the guideline parameters of information exchange were comparatively little addressed during the actual routine care. This might be due to a perceived ‘lack of patient’s interest’ in counselling. Our results suggest to scrutinize whether patients are in fact not interested in counselling and to further explore how the positive intention of pharmaceutical staff towards information exchange can be further translated into everyday practice.

## Introduction

Worldwide self-medication with non-prescription medicine represents a growing issue in the daily counselling practice of community pharmacies [1–5]. However, there are possible risks that can cause harm to the patient such as incorrect self-diagnosis, inappropriate use of non-prescription medication, side effects and interactions with concurrent medication [6–10]. Therefore, consumers who seek access to non-prescription medicines should be supported in their self-medication choices by qualified advisors. It frequently has been reported that appropriate counselling by pharmaceutical staff maximises the benefits and minimises the risks associated with pharmacotherapy [11–15]. Since pharmacies are frequently patients’ first and only point of contact for self-medication [16], they have an important responsibility to ensure patient safety. In Germany, around 19,400 privately owned pharmacies manage self-medication enquiries on a daily basis [5]. In contrast to other countries, most of the non-prescription medications are restricted to be only sold in community pharmacies (pharmacy-only products) [6, 17, 18]. These pharmacy-only products are kept behind the counter out of reach of the patients. As in many other countries, only pharmaceutical staff are allowed to dispense these non-prescription medications. In Germany, pharmaceutical staff consists of pharmacists, pre-approbation trainee pharmacists, pharmaceutical engineers, pharmaceutical technical assistants (PTA) and PTAs in training. Pharmaceutical engineers are a profession that was formerly educated at universities of applied science in the German Democratic Republic (GDR).

To assist pharmaceutical staff in their key role of ensuring patient safety in self-medication, professional organisations worldwide developed good-practice guidelines as a standard for an appropriate and systematic counselling process [19–23]. In Germany, the Federal Chamber of Pharmacists (BAK) developed the German guideline for self-medication consultations [19], which is updated and approved every three years [24]. According to this guideline [19] pharmaceutical staff should initially gather information about the individual patient’s situation. Based on this information pharmaceutical staff should identify potential problems and assist patients by selecting appropriate therapies, and, if needed, referring them to a physician. When a self-care approach is appropriate, pharmaceutical staff should inform the patient about the appropriate use of the medication and about additional health-related considerations [19, 25]. These counselling steps defined for self-medication consultations are mostly in line with those from other developed countries, e.g. in the US [20, 22] and Australia [21, 26]. In summary, guideline-recommended counselling should include pharmaceutical staff exchanging relevant information with the patients to facilitate a safe and appropriate use of self-medication [19, 20].

This exchange of relevant information basically consists of two stages: the information gathering and the provision of information. Gathering information related to the patient’s enquiry is crucial for the pharmaceutical staff to recommend an appropriate therapy and to provide appropriate advice to patients [27, 28]. An increased amount of information exchanged between patients and pharmaceutical staff is associated with positive outcomes of a self-medication consultation, such as a recommendation of appropriate medicine [13, 27, 29, 30]. However, recent evidence suggests that worldwide information exchange during self-medication consultations in the routine care shows areas needing improvement [27, 28, 31–35].

So far, only a few standardized simulated patient studies for a few self-medication indications (i.e. diarrhoea, headache, heartburn, sedating antihistamines and sleeping pills) have been performed in Germany. These studies suggested an insufficient implementation of the guideline-recommendations in the current counselling practice [36–39]. Observed routine data on the information exchange through non-participant observation for a wide range of self-medication topics and for consultations with no scripted customers, however, are scarce so far. Simulated patient visits reflect the performance of a specific staff member at a specific time point with one specific scenario and, therefore, provide only a small picture of the pharmacies’ practice. However, to design appropriate methods to aid the optimisation of the counselling quality during non-prescription medication supply, the current needs in pharmacy services should also be identified through a broader evaluation of the current everyday situation in community pharmacies. Therefore, the aim of this study was to evaluate the current implementation of guideline-recommended information exchange between pharmaceutical staff and customers in German community pharmacies. Firstly, we assessed the attitude towards and the perceived frequency of information exchange as well as the perceived barriers to its implementation from the perspective of the pharmaceutical staff via an online-questionnaire. Secondly, the actual practice was elicited via a non-participant observation of real-life consultations.

## Materials and methods

### Study design and analysed parameters

**Fig 1:**
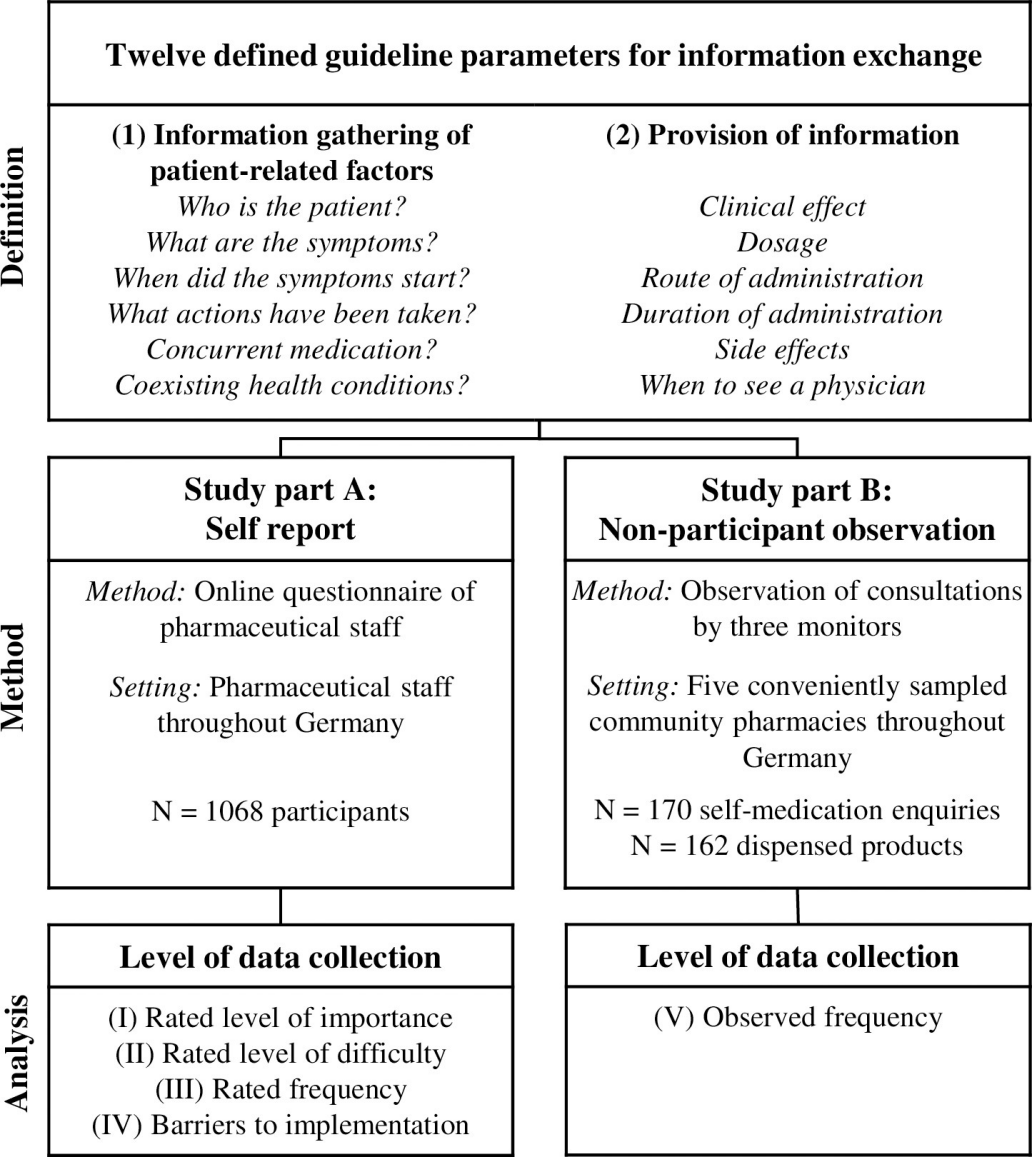
Overview of the two study parts with different levels of data collection and comparison.

A two-part multi-method study was conducted consisting of a (A) self-report and (B) non-participant observation. A consensus group of four pharmacists with vast expertise in healthcare research and counselling patients on self‐medication defined six parameters for gathering patient-related information and six parameters for provision of information (Fig 1). Those parameters were assumed to provide a relevant information exchange when addressed during consultation. In addition, the consensus group defined possible barriers to their implementation. The parameters based on the national guideline for self-medication consultations published by the BAK [19] were complemented with a literature review [20, 22, 40]. These parameters of relevant information exchange were evaluated in both study parts. We invited all professions of pharmaceutical staff (pharmacists, pre-approbation trainee pharmacists, pharmaceutical engineers, PTAs and PTAs in training) to participate in the study in order to obtain a general overview of the current situation in self-medication consultation practice in Germany.

### Study part A: Self-report

#### Participants and setting

We asked all Chambers of Pharmacists in Germany as well as pharmaceutical societies and associations to invite their members to participate voluntarily in an anonymous questionnaire survey to assess the current counselling practice with a special emphasis on the information exchange between pharmaceutical staff and customers. No incentives were offered to participants. The weblink of the survey was distributed via e-mail, social media and in paper form (fax, letter, magazines) depending on the organisation. A precise number of individuals who received the survey is therefore not known. The study’s data set was collected in a two-part online survey together with a study, which evaluated the use of clinical study data in self-medication consultations [41].

The web-based questionnaire survey (SoSci Survey, version 2.6.00-i [42]) was carried out for a five-month period from March 16 to August 15, 2017. It enabled nationwide participation and ensured participants’ privacy as no IP addresses or further data were recorded.

#### Self-administered questionnaire

The structured questionnaire was developed by the consensus group. Four-point Likert scales were used to obtain the participants’ opinions on four levels of data collection: importance, difficulty, rated frequency and barriers to implementation (Fig 1; S1 Appendix).

To confirm feasibility and comprehensibility, the questionnaire was pretested stepwise with 19 pharmacists and pharmaceutical technical assistants (PTA), who were not involved in the development of the study protocol or the survey. The feedback resulted in the final questionnaire. Data from the pretests were not included for final data analysis.

### Study part B: Non-participant observation

#### Participants and setting

Three observers (1 pre-approbation trainee pharmacist, 2 pharmacists) conducted a non-participant observation in five conveniently sampled pharmacies situated in four different federal states (North Rhine-Westphalia, Saxony, Saxony-Anhalt, Thuringia) to assess the actual counselling practices in a real-life setting. All five invited pharmacies and their pharmaceutical staff participated voluntarily in the study. The observer was positioned next to the staff member. The pharmaceutical staff invited the customers to participate voluntarily and asked whether an observer could observe the counselling process and document the counselling anonymously (without any personal data).

Study part B was performed from June 19 to August 17, 2017 with a total of 15 working days of non-participant observation. One to two of the observers conducted observations in each pharmacy. Only consultations in self-medication were analysed. The demographic data of the pharmaceutical staff were recorded through face-to-face interviews.

#### Observation method

An observation form developed by the consensus group consisting of four pharmacists guided the written recording of the consultation as close as possible to the real spoken content. The dialogue was written down in a running text form. In a second step directly after the observation, a quality assurance form (structured coding scheme) helped to determine whether the twelve information exchange parameters (Fig 1) had been fulfilled in the consultation. To ensure the quality of the data, the entry in an electronic sheet was performed on the same day as the observation. The data were reviewed for comprehensibility and the observer’s ratings of the fulfilled information parameters were verified by a pharmacist (one of the two other observers). Thus, at least two persons discussed each consultation process. Incongruent scoring between an observer and the reviewer were resolved by consensus of three reviewers and the observer of the consultation.

To confirm feasibility, the observation sheets were pretested stepwise with audio examples of fictional consultation processes. These examples trained the observers and confirmed that they were writing down the real spoken content. Additionally, we pretested for one observation day in a pharmacy with real consultation processes. The data of the pretests were not included in the data analysis of the main study.

### Data analysis

The data analysis for both study parts was performed using Microsoft Office Excel 2016 (Microsoft Corporation, Redmond, Washington, USA) and IBM SPSS Statistics Version 25.0 (IBM Corporation, Armonk, New York, USA). Continuous data were presented as mean with standard deviation (SD) or as median with first and third quartile (Q25/75) and minimum and maximum (Min/Max) depending on the distribution. To test for normality in distribution, the Shapiro Wilk test was used. Nominal and ordinal data were reported with absolute numbers (N) and relative (%) frequencies. Missing data are described as “not specified”. Pharmaceutical staff who reported to consider the twelve parameters in the self-report in most or almost all consultations were considered to “address them in routine counselling”. Addressing the parameters in a few or hardly any consultation was considered “not addressing them in routine care". In the observation, the written notes of the observed dialogues were assessed for the twelve defined parameters of information exchange. If pharmaceutical staff or the customer addressed at least one aspect of an information parameter (e.g. one concurrent medication) this parameter was defined as fulfilled (dichotomous scoring system for every parameter). The statistical analysis for the ratings and performance of pharmaceutical staff with university degree and without university degree was performed with a Mann-Whitney U test for unpaired data (without normal distribution). Pharmaceutical staff with a university degree included pharmacists, pre-approbation trainee pharmacists and pharmaceutical engineers. Staff members without university degree consisted of PTA and PTA in training. The z-values were used to calculate the Pearson correlation coefficient |r| as effect size of the Mann-Whitney U test. A value of |r| = 0.10–0.29 is considered as small, |r| = 0.30–0.49 as medium and |r| ≥ 0.5 as large correlation [43]. The threshold for statistical significance was set at p < 0.05.

### Ethics

The self-administered online questionnaire in pharmaceutical staff was anonymous. The self-medication consultations were observed under routine conditions as a part of the quality assurance strategy of the pharmacies. During the observation of this existing pharmacy service, no personal identifiable customer information was collected. Therefore, the Ethics Committee of the Medical Faculty of Leipzig University confirmed that according to the German legal requirements in terms of § 15 of the code of medical ethics from the State Chamber of Physicians of Saxony no ethical review was required for this research project. The responsible Chambers of Pharmacists were informed about the study in advance. All work was conducted in accordance with the principles of the Declaration of Helsinki. Participation in the study was on a voluntary basis. Each participant was informed on the objectives of the study and asked beforehand about his or her willingness to take part. Consent to participate in the anonymous questionnaire survey was assumed by the completion of the survey. For the observation of routine self-medication consultations, customers gave informed verbal consent and pharmaceutical staff gave written informed consent.

## Results

### Study part A: Self-report

#### Characteristics of participants

In total, 1068 members of the pharmaceutical staff in community pharmacies throughout Germany completed the survey. When considering a total of about 124 000 pharmaceutical staff members in community pharmacies, this corresponds to a sample of around 1% of all staff eligible to counsel on non-prescription drugs throughout Germany. The participants were predominantly female (78%) and claimed to be involved frequently in self-medication counselling. The median work experience in community pharmacies of the participants was 15 years (Q25/75: 6/25, Min/Max: 0/50). Table 1 shows the demographic data of the participants.

**Table 1:** Characteristics of participants in the self-report (study part A) [N total = 1068].

Characteristics	Values
**Median age** [years (Q25/Q75; Min/Max)]	41 (31/51; 20/80)^a^
Not specified [N (%)]	4 (0%)
**Gender female** [N (%)]	831 (78%)
Not specified [N (%)]	3 (0%)
**Profession**	
Pharmacist [N (%)]	846 (79%)
Pre-approbation trainee pharmacist [N (%)]	46 (4%)
Pharmaceutical engineer [N (%)]	12 (1%)
Pharmaceutical technical assistant [N (%)]	163 (15%)
Not specified [N (%)]	1 (0%)
**Median work experience in the community pharmacy** [years (Q25/Q75; Min/Max)]	15 (6/25; 0/50) ^a^
Not specified [N (%)]	4 (0%)
**Median weekly working time in the community pharmacy** [hours (Q25/Q75; Min/Max)]	40 (30/40; 1/80) ^a^
Not specified [N (%)]	4 (0%)
**Frequency of activity in counter sales**	
Always [N (%)]	346 (32%)
Frequently [N (%)]	628 (59%)
Sometimes [N (%)]	79 (7%)
Seldom [N (%)]	12 (1%)
Never [N (%)]	0 (0%)
Not specified [N (%)]	3 (0%)

*Q25*: first quartile; *Q75*: third quartile; Min: minimum; Max: maximum.

The rounding of values may result in total amounts deviating from 100%.

^a^ 1 value excluded due to plausibility of the value.

#### Level of importance (I) and level of difficulty (II)

**Fig 2:**
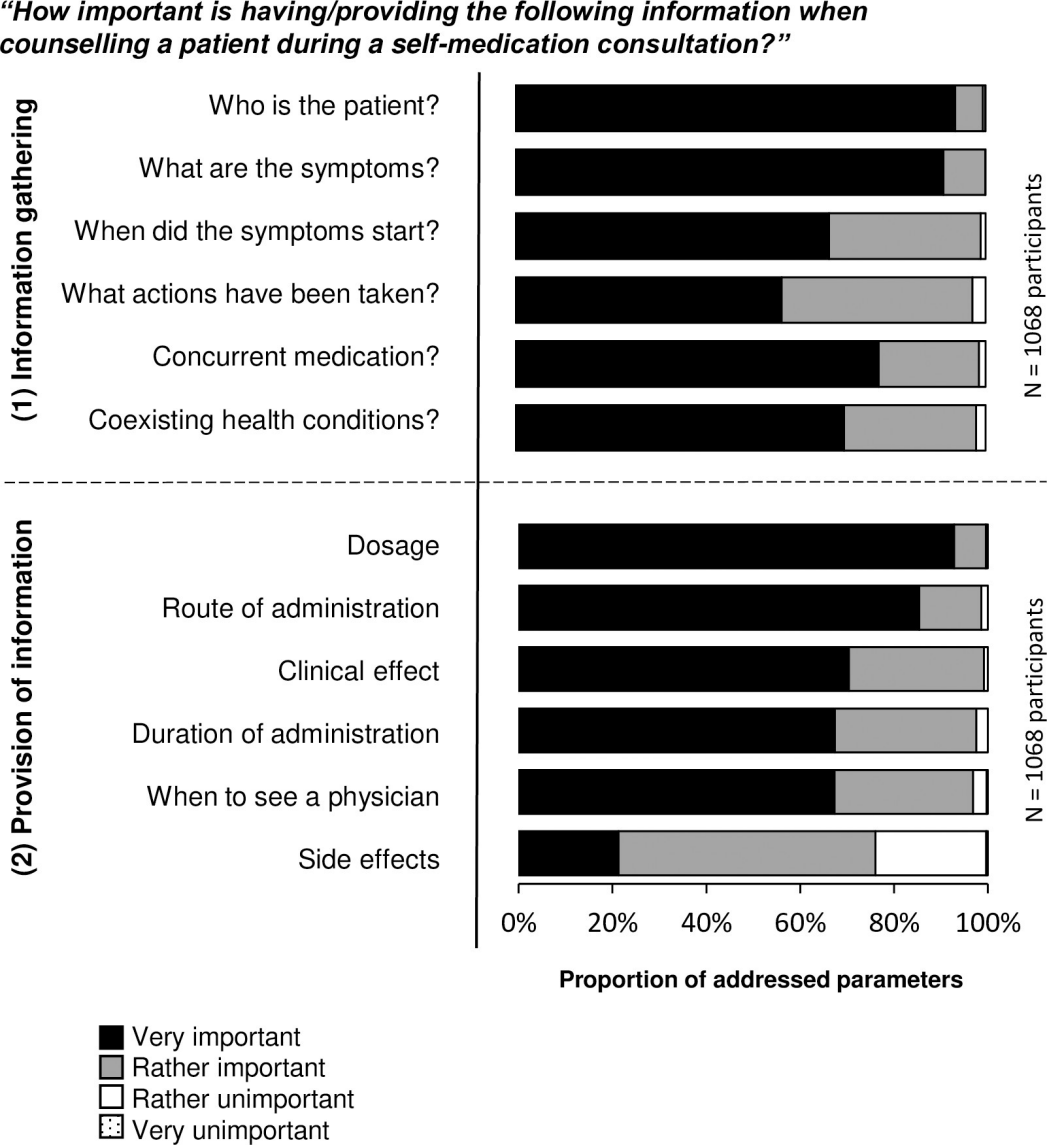
Self-reported levels of importance (I) of the parameters during a self-medication consultation. The twelve parameters were rated by 1067 respondents in the self-report (study part A). One participant did not specify the importance of the parameters.

**Fig 3:**
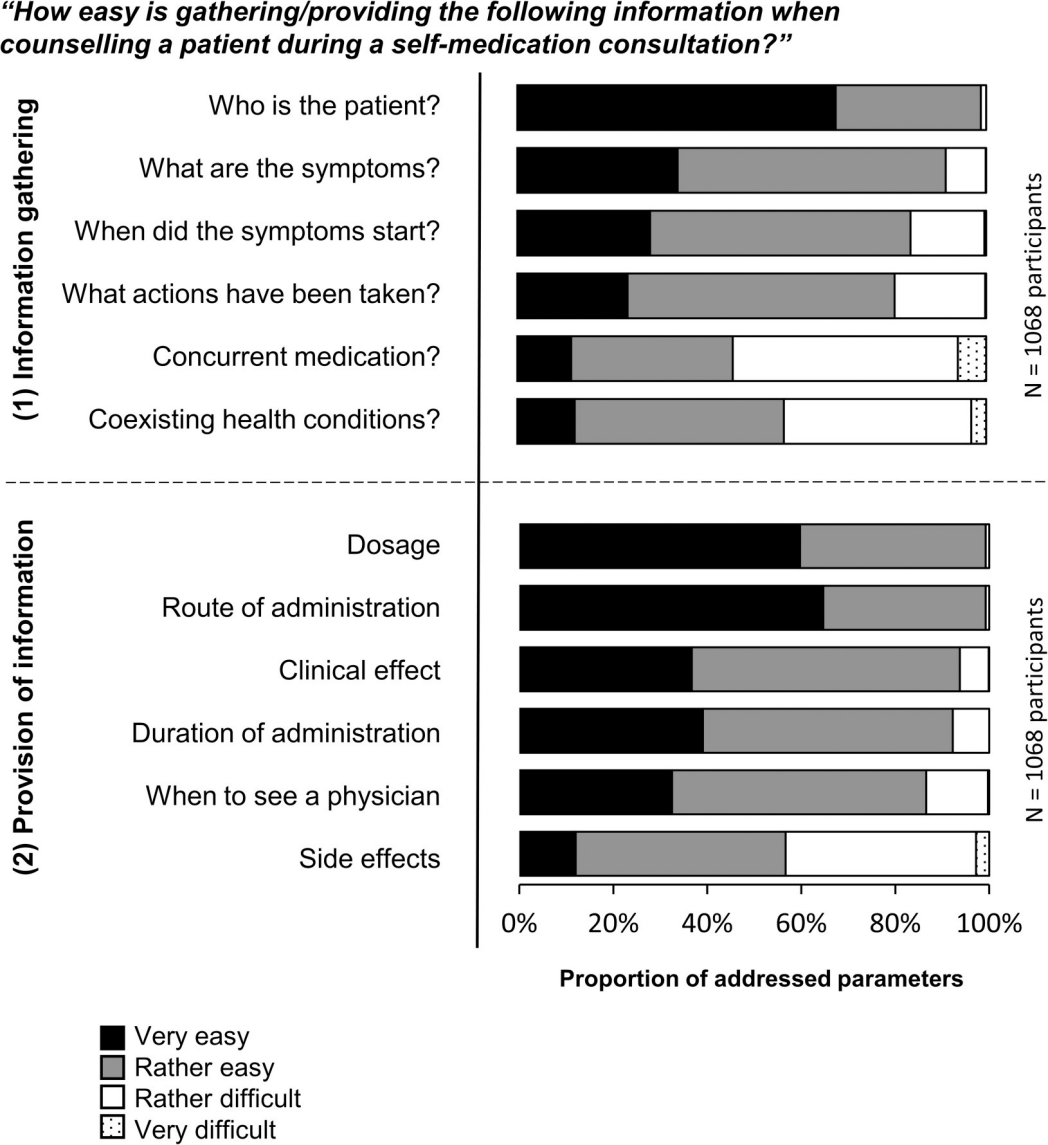
Self-reported levels of difficulty (II) of the parameters during a self-medication consultation. The twelve parameters were rated by 1068 respondents in the self-report (study part A).

Every parameter of information gathering during self-medication consultations was rated by more than 97% of the participants as very or rather important (Fig 2). Gathering the patient information on ‘co-existing health conditions’ and ‘concurrent medication’ during a patient counselling process was perceived as difficult by 43% [460/1068] and 54% [576/1068] of the participants, respectively (Fig 3).

All parameters but one for providing information were rated by more than 96% of the participants as important (Fig 2). Hence, the parameter ‘side effects’ was least likely to be rated as important by the respondents (76% [811/1067]). Besides being the least important parameter, ‘side effects’ was most likely to be determined as a difficult parameter to provide information about (44% [463/1068]; Fig 3).

#### Rated frequency (III) of addressing the parameters

**Fig 4:**
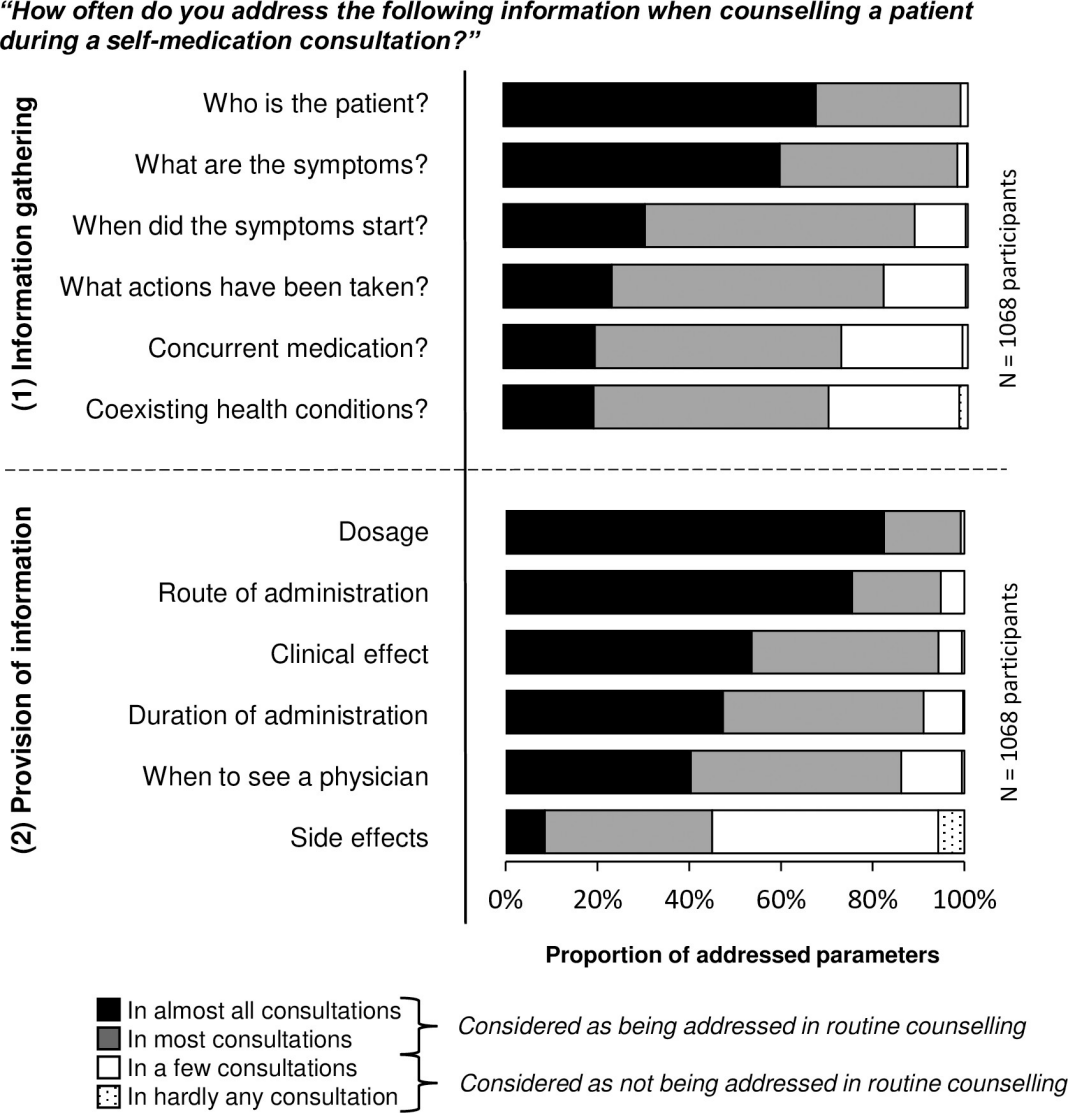
Rated frequency (III) of addressing the twelve counselling parameters during a self-medication consultation. The twelve parameters were rated by 1068 respondents in the self-report (study part A).

According to at least 82% of the participants in the self-report, information was addressed during routine counselling processes on ‘who is the patient’, ‘what are the symptoms’, ‘when did the symptoms start’ and ‘what actions had been taken’. Information on ‘concurrent medication’ and ‘coexisting health conditions’ was supposedly collected routinely by only 73% [777/1068] and 70% [748/1068] of the participants in routine counselling, respectively (Fig 4). The ratings of the quantity of fulfilled information parameters for information gathering were statistically significantly higher for pharmaceutical staff with a university degree than for staff without university degree (Mann-Whitney U test: n = 1067; Z = -2.374; p = 0.018; r = 0.073).

At least 86% of pharmaceutical staff claimed that during routine counselling processes, they provide information about ‘dosage’, ‘route of administration’, ‘clinical effect’, ‘duration of administration’ and ‘when to see a physician’. Fewer than half of the pharmaceutical staff stated that they address information about ‘side effects’ in routine counselling (45% [481/1068]; Fig 4). The ratings of the quantity of fulfilled information parameters in routine counselling for the provision of information did not differentiate between staff with and without university degree (Mann-Whitney U test: n = 1067; Z = -1.172; p = 0.241; r = 0.036).

#### Barriers to implementation (IV)

**Fig 5:**
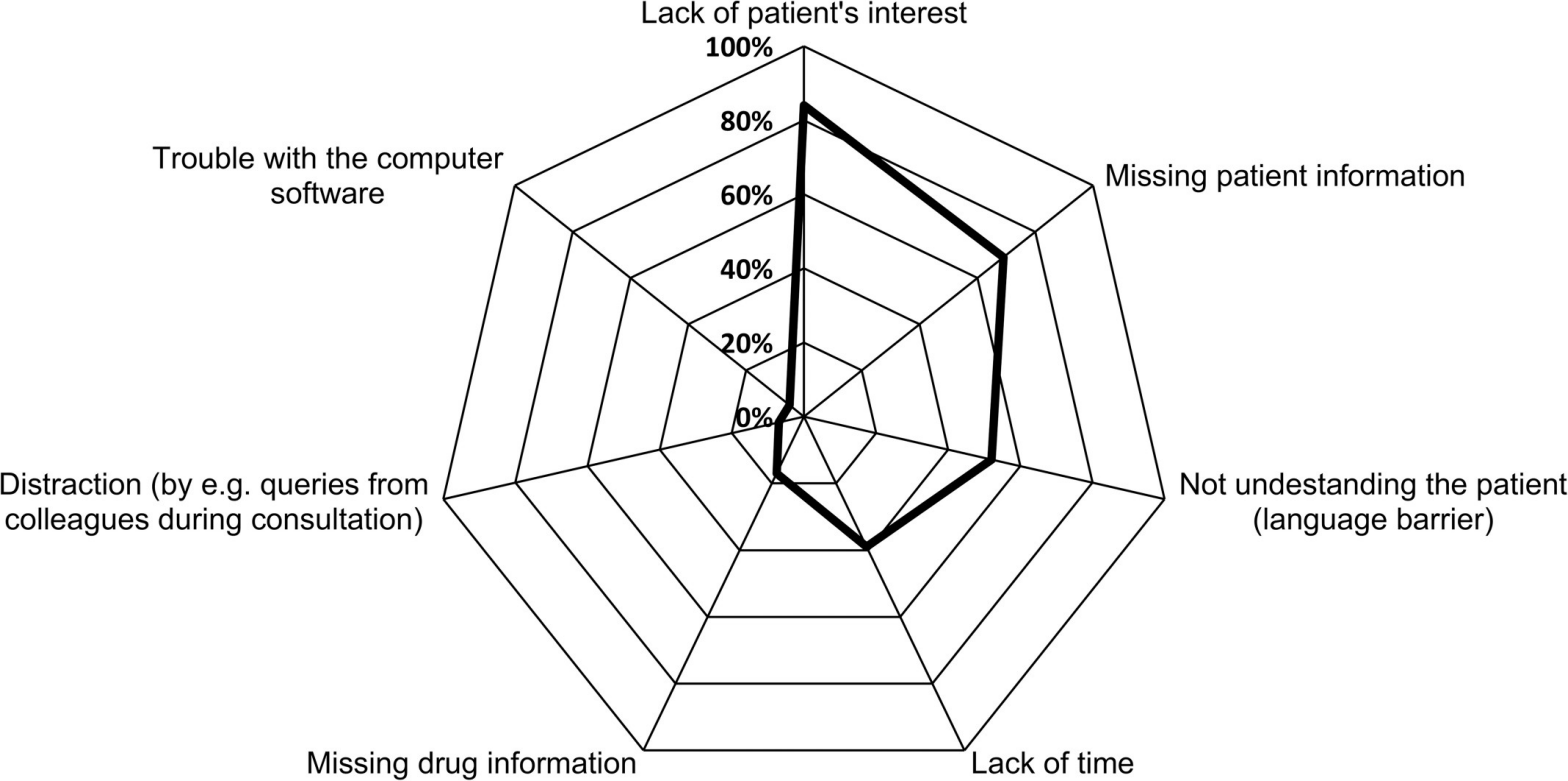
Pharmaceutical staff’s view of barriers to an appropriate counselling process. Answers to the question “Which of the following factors prevents you from providing an optimal patient consultation for self-medication?” [Multiple choice; N (total) = 1068].

When asked about barriers preventing the pharmaceutical staff from giving appropriate patient counselling in self-medication, 1016 of 1068 respondents (95%) stated at least one of seven predefined factors (Median: 3; Q25/75: 2/3; Min/Max: 0/7). It is apparent in Fig 5 that patient factors such as ‘lack of patient’s interest’ (84% [891/1068]) and ‘missing patient information’ (69% [739/1068]) are most frequently cited as hindering the pharmaceutical staff from providing optimal counselling.

### Study part B: Non-participant observation

#### Characteristics of participants and analysed processes

In total, 24 pharmaceutical staff members were observed in the five pharmacies. All of them were female (100%) with a median of 13 years of work experience (Q25/75: 5/24, Min/Max: 2/41) in community pharmacies. The majority stated that they were always (38%) or frequently (46%) involved in self-medication counselling. Table 2 provides the demographic data of the participants. 108 consultations with 170 self-medication enquiries were included. These enquiries resulted in dispensing a total of 162 self-medication products. The median of observed consultations per community pharmacy were 19 (Q25/75: 13/22; Min/Max: 11/43). The median of self-medication enquiries per community pharmacy were 31 (Q25/75: 17/41; Min/Max: 15/66). The number of self-medication enquiries observed for each participating member of pharmaceutical staff ranged from minimum 1 to maximum 24 (Median: 5; Q25/75: 3/10).

**Table 2:** Characteristics of participants in the non-participant observation (study part B) [N total = 24].

Characteristics	Values
**Mean age** [years (± SD)]^a^	36.67 (±2.06)
**Gender female** [N (%)]	24 (100%)
**Profession**	
Pharmacist [N (%)]	5 (21%)
Pre-approbation trainee pharmacist [N (%)]	1 (4%)
Pharmaceutical engineer [N (%)]	3 (13%)
Pharmaceutical technical assistant [N (%)]	15 (63%)
**Median work experience in the community pharmacy** [years (Q25/Q75; Min/Max)]^b^	13 (5/24; 2/41)
**Median weekly working time in the community pharmacy** [hours (Q25/Q75; Min/Max)]^b^	38 (30/40; 15/45)
**Frequency of activity in counter sales**	
Always [N (%)]	9 (38%)
Frequently [N (%)]	11 (46%)
Sometimes [N (%)]	3 (13%)
Seldom [N (%)]	1 (4%)
Never [N (%)]	0 (0%)

*Q25*: first quartile; *Q75*: third quartile; Min: minimum; Max: maximum.

The rounding of values may result in total amounts deviating from 100%.

^a^ Data with normal distribution. For purposes of comparison the median age was 34 (Q25/Q75: 27/44; Min/Max: 25/59).

^b^ Data without normal distribution.

#### Observed frequency (V) of addressing the parameters

**Fig 6:**
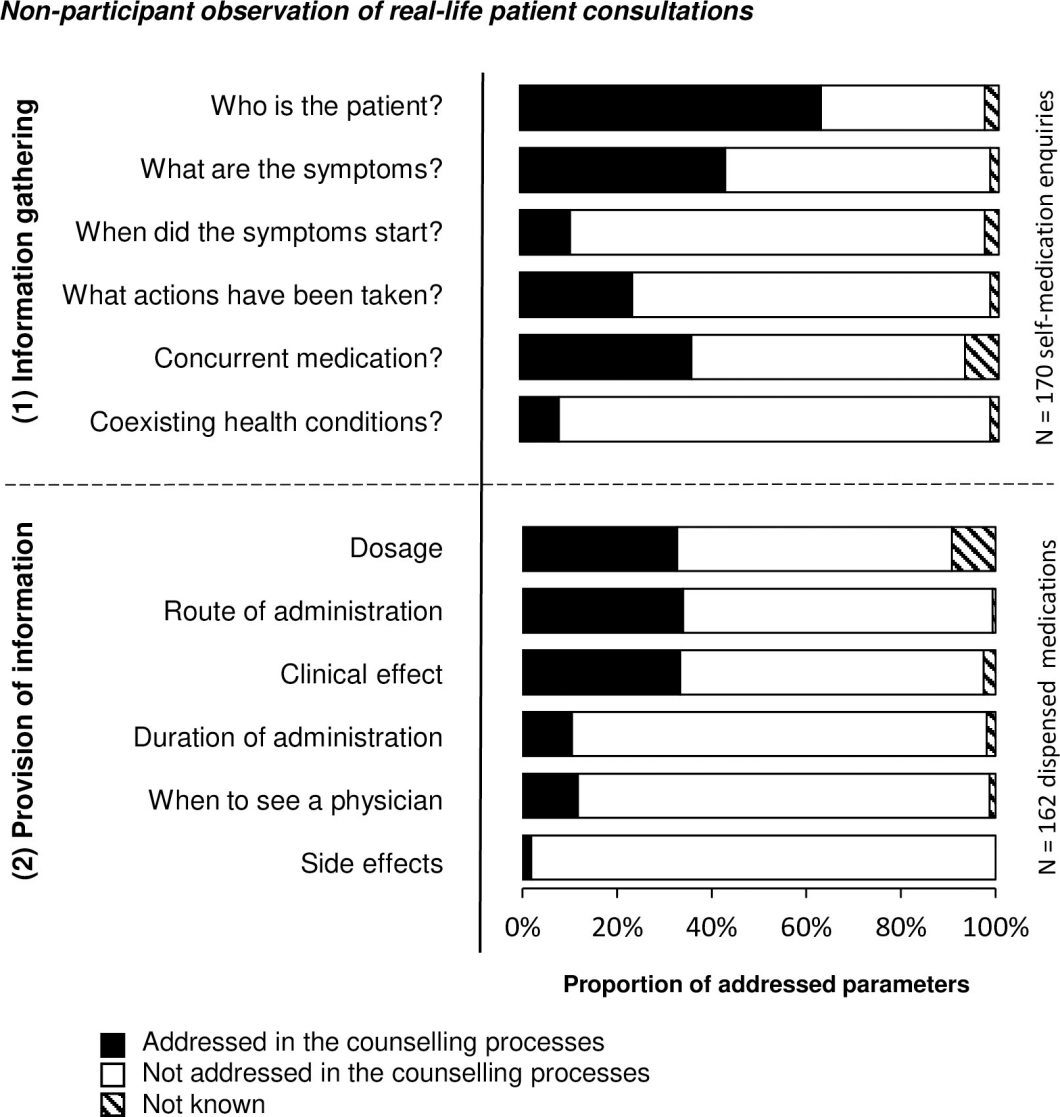
Observed frequency (V) of addressing the twelve parameters in the observation (study part B). 170 observed real-life self-medication enquiries were evaluated for the observed frequency (V) of addressing the parameters of information gathering (1). In 162 (100%) dispensed product processes the six parameters of information provision (2) were assessed.

The non-participant observation showed that the majority of pharmaceutical staff did not routinely gather information about the six patient-related parameters. The most common types of information gathered by pharmaceutical staff in the 170 self-medication enquiries were ‘who is the patient’ (63% [107/170]) and ‘what are the symptoms’ (43% [73/170]; Fig 6). We found no statistical difference in the observed number of information parameters gathered by pharmaceutical staff member with versus without a university degree (Mann-Whitney U test: n = 170; Z = -0.550; p = 0.582; r = 0.042).

In the provision of information, the results revealed that the customers were not routinely informed about the six parameters during the 162 non-prescription product sales. Information on ‘side effects’ (2% [3/162]) was the least common type of information provided (Fig 6). The quantity of information parameters provided did not differentiate between pharmaceutical staff members with and without a university degree (Mann-Whitney U test: n = 170; Z = -0.627; p = 0.531; r = 0.048).

## Discussion

We examined the information exchange in a multi-method assessment (self-report and non-participant observation) to evaluate both the self- and external perception of the current implementation of guideline-recommended information exchange during self-medication consultations. The results of the self-report showed that pharmaceutical staff considered the guideline parameters as important. Most of the parameters were rated easy to address and the participants reported addressing these parameters during self-medication consultations. However, the non-participant observation revealed suboptimal implementation of the guideline parameters in the actual counselling practice. Thus, the findings suggest that despite broad acceptance of the counselling guidelines by the pharmaceutical staff, the parameters have not yet been fully integrated into everyday practice.

### Perceptions about the information exchange

In the self-report, the pharmaceutical staff acknowledged all of the twelve guideline parameters for information exchange during self-medication consultation and reported to have implemented them during routine counselling practice.

For information gathering, the parameters rated the most difficult to fulfil were also rated to be addressed the least. Questions about ‘coexisting health conditions’ and ‘concurrent medication’ might be difficult to address because patients often cannot provide information about these parameters [44, 45] and no electronic patient files are available for pharmacies in Germany at this point in time. For some patients, relevant information about the dispensing history or allergies are documented in a pharmacy when they have a loyalty card of the pharmacy. However, this information cannot be shared between health care professionals and pharmaceutical staff might be reluctant to ask questions that seem too personal [46, 47].

For the provision of information, the parameter ‘side effects’ was the least commonly reported as important, the most difficult and also the least addressed parameter. According to other studies, this could originate from pharmacists’ belief that information about side effects could hinder the patients from taking their medications [48]. They do not want to compromise the adherence [48] and their relationship with their customers [35]. Moreover, it might be possible that they fear a loss of revenues in self-medication sales. Nevertheless, to assure patient safety, patients should receive the information about the potential risks of their drugs and the appropriate handling of potential side effects. Patients even expect this information from their health care professionals [48–50]. Therefore, the more crucial question should be what is a safe and accessible way to inform patients about relevant information such as side effects.

### Information exchange in actual practice

In the non-participant observation, we found suboptimal implementation of the twelve guideline parameters. According to our sample of observed processes gathering patient information and providing information has not yet been fully integrated into the everyday practice. When comparing our results with an international observation study from Watson et al. [29] the information on ‘who is the patient’ was gathered in our study in less consultations (76% vs. 63%). During simulated patient studies performed in Germany, gathering information on ‘who is the patient’ ranged even between 37% and 92% of the consultations [36, 37, 39, 51]. The information on ‘symptoms’ was almost similar frequently provided in Watson et al.’s study [29] compared to our study (37% vs. 43%). The information on ‘dosage’ ranged in international studies between 16% to 97% [52–54] compared to 33% in our study. In German simulated patient studies, the provision of the parameter ‘dosage’ also differed considerably between the conducted studies (18% vs. 56% vs. 87% vs. 89%) [36, 37, 39, 51]. This could possibly result from the different underlying simulated case scenarios.

Generally, self-medication guidelines provide a framework for an appropriate information exchange. Due to the complex mechanism of interaction, however, guideline implementation in daily practice is challenging [55]. The feasibility of the current guidelines should therefore be questioned and optimized by providing more guidance on prioritising the guideline parameters for specific patient situations in everyday practice.

Although evidence is humble, training provides a promising strategy to improve skills and attitudes regarding guideline implementation [40, 56]. A structured interview framework simplifies remembering guideline parameters and can improve counselling skills [40]. E-learning and Objective Structured Clinical Examinations (OSCE) as an examination tool proved to be successful methods to improve pharmacy consultations in chronic diseases [56]. Communication skills training has been shown to improve the communication competency of students [57]. Although defined as pharmacist’s expertise [58], communication training is still not a mandatory part in German university education [59]. Compared to other European countries, more time is still dedicated to chemical science courses than to medicinal science courses [60]. However, it must be noted that pharmacy courses have become more ‘clinical’ in recent years and therefore consultation skills are more likely to be found in the syllabus. Nevertheless, communication training for patient’s consultations is officially part of the practical trainee year after university education [59]. Thereby the quality of the training greatly depends on the single teaching pharmacy. Thus, the implementation of such tailored training programs might be useful to improve information exchange in self-medication counselling.

### Perceived barriers to information exchange

Pharmaceutical staff noted that the main impediment for appropriate counselling was ‘lack of patient’s interest’. This is in line with literature reporting the ‘lack of patient’s interest’ as barrier for the counselling practice [61, 62]. Patients are more focused on buying the product than on obtaining professional advice [46]. They might have made their own decision for a product before entering the pharmacy [63]. This could lead to patients being less interested in counselling although their own assessment might be wrong and a potential risk for their health. Moreover, patients might be reluctant to ask questions out of fear or embarrassment of seeming uneducated [64]. Pharmaceutical staff could support customers’ interest and engagement by asking relevant questions, since this has been shown to trigger patients to provide information [65]. Further research needs to explore whether the patients are indeed not interested in appropriate counselling and what strategies can lead to their further engagement in consultations.

### The divergence between perception and practice as a potential barrier for optimisation

The results of our study indicate that there might be a difference between pharmaceutical staff’s reported perceptions and their actual practice. Hence, this represents a possible barrier to implement self-medication guidelines in everyday counselling practice. Pharmaceutical staff intended to counsel in adherence to the guidelines and thought that they are already doing it (‘reported practice’). However, the observation (‘actual practice’) showed that the information parameters have not been completely implemented. This discrepancy between work as rated (self-report) and work as done (observed) is in line with the international literature [27, 29, 52, 66–68] and has been described for developed as well as developing countries, e.g., the UK, Northern Cyprus, Indonesia and Ethiopia [27, 29, 52, 66]. A reason for this, in addition to the feasibility of the guidelines, could be that intentions do not always translate into practice [69, 70]. As long as there is no awareness of the need for optimisation, it will be difficult to encourage pharmaceutical staff to change their behaviour.

### Further implications for practice

We suggest that both, the guideline and the behaviour of the pharmaceutical staff, needs to be adapted to real-life practice. Guidelines should better incorporate recommendations for implementation or prioritisation of information exchange parameters in the actual practice. Strategies to promote self-reflection by pharmaceutical staff should be developed to modify their behaviour. Thereby, the divergence between pharmaceutical staff’s reported perceptions and their actual practice as well as the management of patient’s lack of interest in counselling should be considered. Besides further investigations of influencing factors for information exchange, patient-centred practical training in counselling should be a mandatory regular element in continuing education of pharmaceutical staff. This could further foster the use of the guideline parameters in the actual practice.

### Limitations

Since data in the self-report were collected anonymously, targeted reminders to non-respondents could not be sent. An overestimation due to social desirability bias and a participation of those who were particularly interested in the topic cannot be excluded. Participants were asked about general perceptions of their everyday counselling practice. Nevertheless, this perception could differ in respect to specific situations (e.g. customers with many co-existing health conditions). Due to an expected social desirability bias, we could not evaluate a potential ‘lack of interest’ by pharmaceutical staff as a barrier to counselling. It is noticeable that more pharmacists than PTA were involved in the self-report. In the observation, it was the other way around. This reflects the reality in Germany where pharmacists are responsible for modalities of the counselling in the pharmacy and PTA actually provide many of the counselling processes (under the supervision of a pharmacist). Nevertheless, this fact may cause differences in the observed practice with respect to other countries.

The observation of the participants may have influenced the counselling practice of pharmaceutical staff to ‘do their very best’ (Hawthorne effect). However, studies found that observers of others’ behaviour can have high validity, especially if those observed behaviours are carefully defined [71, 72]. The observation of consultations was a point-in-time measurement and, therefore, did not consider potential influencing factors such as seasonal effects. Moreover, it was not considered how busy the pharmacy was, e.g. like counting the current customers in the pharmacy. We chose non-participant observations since we wanted to elicit real-life consultations on a wide range of self-medication topics and gain a broad overview of the current everyday situation in community pharmacies. This limited the number of observed community pharmacies (n = 5) compared to the use of a simulated patient methodology, which would have allowed the inclusion of more pharmacies but less staff members and limited indications in scripted scenarios. To gain the most realistic picture of the consultation practice, both complementary methods should be used for research in self-medication consultations. Even though we chose pharmacies from different regions for the non-participant observation, the limited number of conveniently sampled pharmacies and observed processes require caution when making generalisations with respect to the results of the real-life counselling practice. Since these pharmacies were open to have their practice observed it cannot be excluded that they were more professionally-oriented than others.

Because of the anonymity of the survey it is not known, whether the pharmacies in the observation also participated in the survey. As a consequence of the different methods of data collection in the two study parts, a statistical comparison of the results was not applicable. The patients’ perception was not targeted in this study but has already been evaluated by our research group [73].

## Conclusion

Despite broad acceptance of the counselling guidelines by the pharmaceutical staff, the information exchange parameters have not yet been fully integrated into the everyday pharmacy practice. One reason for this could be that the pharmaceutical staff perceived a lack of patient’s interest as a major barrier to their counselling. To improve drug safety there is a need to further investigate why guideline parameters are not sufficiently addressed during consultations and to explore whether patients really lack in interest for self-medication counselling.

## Supporting information

S1 Appendix Structure of the questionnaire. The original questions of the self-report (study part A) and the answering scales are presented.
(PDF)

S1 Data Values behind the means, medians, graphs and other measures of the manuscript. (XLSX)
