# Supplementary material for: What is the attitude towards and the current practice of information exchange during self-medication counselling in German community pharmacies? An assessment through self-report and non-participant observation
Source: PLoS One. 2020 Oct 14;15(10):e0240672. doi: 10.1371/journal.pone.0240672 (PMC7556488; doi:10.1371/journal.pone.0240672)
Supplement: S1 Appendix — The original questions of the self-report (study part A) and the answering scales are presented. (PDF) [file pone.0240672.s001.pdf]

What is the attitude towards and the current practice of information exchange during self-medication counselling in German community pharmacies? An assessment through self-report and non-participant observation

Seiberth JM, Moritz K, Kücükay N, Schiek S, Bertsche T

Drug Safety Center, University Hospital Leipzig and Leipzig University and Department of Clinical Pharmacy, Institute of Pharmacy, Faculty of Medicine, Leipzig University, Leipzig, Germany;
E-mail to thilo.bertsche@uni-leipzig.de

S1 Appendix:

Structure of the questionnaire. The original questions of the self-report (study part A) and the answering scales are presented.

Level of data collection	Requested information
**Level of importance** of information gathering and provision of information during self-medication counselling	**Importance** of the 6 parameters for **information gathering**:<br>*"How important is having the following information when counselling a patient during a self-medication consultation?"*<br>(1) 'Who is the patient?', (2) 'What are the symptoms?', (3) 'When did the symptoms start?' (4) 'Which actions have been taken?', (5) 'Coexisting health conditions?', (6) 'Concurrent medication?'<br>**Importance** of the 6 parameters for the **provision of information**:<br>*"How important is it for the patient to get the following information when buying medication for self-medication?"*<br>(7) 'Clinical effect', (8) 'Dosage', (9) 'Route of administration', (10) 'Duration of administration', (11) 'Side effects', (12) 'When to see a physician'<br><br>All parameters were rated with 4-point Likert scales: very important, rather important, rather unimportant, very unimportant
**Level of difficulty** of information gathering and provision of information during self-medication counselling	**Level of difficulty** to ask the 6 parameters for **information gathering**:<br>*"How easy is gathering the following information when counselling a patient during a self-medication consultation?"*<br>(1) 'Who is the patient?', (2) 'What are the symptoms?', (3) 'When did the symptoms start?' (4) 'Which actions have been taken?', (5) 'Coexisting health conditions?', (6) 'Concurrent medication?'<br>**Level of difficulty** to provide the 6 parameters for the **provision of information**:<br>*"How easy is it to provide the following information when selling medication for self-medication?"*<br>(7) 'Clinical effect', (8) 'Dosage', (9) 'Route of administration', (10) 'Duration of Administration', (11) 'Side effects', (12) 'When to see a physician'<br><br>All parameters were rated with 4-point Likert scales: very easy, rather easy, rather difficult, very difficult
**Practical procedure** of information gathering and provision of information during self-medication counselling (frequency of the parameters)	**Self-reported frequency** of considering the 6 parameters for **information gathering**:<br>*"How often do you address the following information when counselling a patient during a self-medication consultation?"*<br>(1) 'Who is the patient?', (2) 'What are the symptoms?', (3) 'When did the symptoms start?' (4) 'Which actions have been taken?', (5) 'Coexisting health conditions?', (6) 'Concurrent medication?'<br>**Self-reported frequency** of explaining the 6 parameters for the **provision of information**:<br>*"How often do you provide the following information when selling medication for self-medication?"*<br>(7) 'Clinical effect', (8) 'Dosage', (9) 'Route of administration', (10) 'Duration of Administration', (11) 'Side effects', (12) 'When to see a physician'<br><br>All parameters were rated with 4-point Likert scales: in almost all, in most, in a few or in hardly any consultations [Scales were dichotomized to "addressed in routine counselling" (in almost all/most consultations) and "not addressed in routine counselling" (in a few/hardly any consultations)]

What is the attitude towards and the current practice of information exchange during self-medication counselling in German community pharmacies? An assessment through self-report and non-participant observation

Seiberth JM, Moritz K, Kücükay N, Schiek S, Bertsche T

Drug Safety Center, University Hospital Leipzig and Leipzig University and Department of Clinical Pharmacy, Institute of Pharmacy, Faculty of Medicine, Leipzig University, Leipzig, Germany;
E-mail to thilo.bertsche@uni-leipzig.de

**Barriers** to counselling	**Self-reported frequency of barriers** to counselling processes*:<br>*"Which of the following factors prevents you from providing an optimal patient consultation for self-medication?"*<br>"Often prevents me, that …<br>(1) "… I don't have enough time."<br>(2) "… the patient has no interest in my counselling."<br>(3) "… I don't know enough about the patient."<br>(4) "… I don't understand the patient."<br>(5) "… I am distracted during the consultation."<br>(6) "… I have struggles with the computer software."<br>(7) "… I don't have enough information about the medication."

* The counselling process combines information gathering of the patient and providing information to the patient.
